# The RNAi machinery controls distinct responses to environmental signals in the basal fungus *Mucor circinelloides*

**DOI:** 10.1186/s12864-015-1443-2

**Published:** 2015-03-25

**Authors:** Francisco E Nicolás, Ana Vila, Simon Moxon, María D Cascales, Santiago Torres-Martínez, Rosa M Ruiz-Vázquez, Victoriano Garre

**Affiliations:** Department of Genetics and Microbiology, Faculty of Biology, University of Murcia, 30100 Murcia, Spain; The Genome Analysis Centre, Norwich, NR4 7UH UK

**Keywords:** Asexual sporulation, Sexual interaction, pH regulation, Non-canonical RNAi pathway, esRNAs, mRNA profiling

## Abstract

**Background:**

RNA interference (RNAi) is a conserved mechanism of genome defence that can also have a role in the regulation of endogenous functions through endogenous small RNAs (esRNAs). In fungi, knowledge of the functions regulated by esRNAs has been hampered by lack of clear phenotypes in most mutants affected in the RNAi machinery. Mutants of *Mucor circinelloides* affected in RNAi genes show defects in physiological and developmental processes, thus making *Mucor* an outstanding fungal model for studying endogenous functions regulated by RNAi. Some classes of *Mucor* esRNAs map to exons (ex-siRNAs) and regulate expression of the genes from which they derive. To have a broad picture of genes regulated by the silencing machinery during vegetative growth, we have sequenced and compared the mRNA profiles of mutants in the main RNAi genes by using RNA-seq. In addition, we have achieved a more complete phenotypic characterization of silencing mutants.

**Results:**

Deletion of any main RNAi gene provoked a deep impact in mRNA accumulation at exponential and stationary growth. Genes showing increased mRNA levels, as expected for direct ex-siRNAs targets, but also genes with decreased expression were detected, suggesting that, most probably, the initial ex-siRNA targets regulate the expression of other genes, which can be up- or down-regulated. Expression of 50% of the genes was dependent on more than one RNAi gene in agreement with the existence of several classes of ex-siRNAs produced by different combinations of RNAi proteins. These combinations of proteins have also been involved in the regulation of different cellular processes. Besides genes regulated by the canonical RNAi pathway, this analysis identified processes, such as growth at low pH and sexual interaction that are regulated by a *dicer*-independent non-canonical RNAi pathway.

**Conclusion:**

This work shows that the RNAi pathways play a relevant role in the regulation of a significant number of endogenous genes in *M. circinelloides* during exponential and stationary growth phases and opens up an important avenue for in-depth study of genes involved in the regulation of physiological and developmental processes in this fungal model.

**Electronic supplementary material:**

The online version of this article (doi:10.1186/s12864-015-1443-2) contains supplementary material, which is available to authorized users.

## Background

RNA silencing or RNA interference (RNAi) is a conserved defence mechanism against invasive nucleic acids, such as viruses, transposons or transgenes, in a wide spectrum of eukaryotic organisms, including fungi [1,2]. Double stranded RNA (dsRNA) molecules, derived from those exogenous sequences, are processed by the RNase III Dicer to produce short interfering RNAs (siRNAs), which are bound to an Argonaute protein within the RNA-induced silencing complex (RISC). These siRNAs serve as a guide to identify complementary target RNA molecules for silencing or destruction [3,4]. In many organisms, siRNA-induced silencing requires RNA-dependent RNA polymerases (RdRPs) to generate dsRNA from single-stranded RNA (ssRNA) or to amplify siRNA signals [5]. In metazoans, RNAi also has a role in the regulation of endogenous functions through several classes of endogenous small RNA (esRNAs) molecules, which are generated from genome-encoded precursors. These endogenous pathways play many fundamental roles, including regulation of mRNA accumulation and translation, chromatin silencing, programmed DNA rearrangements and genome surveillance [4]. Different classes of regulatory esRNAs have been also described in fungi, although information on their functional roles is very scarce [2].

*Mucor circinelloides*, a basal fungus belonging to the order Mucorales, has become a good model organism for the study of different molecular processes in the fungal kingdom, including light responses [6] and gene silencing [5], mainly due to the availability of a large number of molecular tools and to its evolutionary distance from other fungal model organisms, such as *Neurospora crassa*. More recently, *M. circinelloides* is attracting special attention as a causal agent of mucormycosis, an emerging fungal infection, not very common but often lethal, caused by various species of the order Mucorales [7]. Although typically affects immunocompromised patients [8], various circumstances such as the use of certain antifungal drugs [9] and some natural disasters [10] have increased the number of cases in risk populations and have boosted the interest in further study about pathogenesis of this fungus [11-13]. Of particular interest is the recent discovery of a new epigenetic mechanism for developing transient resistance to an antifungal drug via an RNAi-mediated pathway, this epigenetic mechanism being particularly enhanced in pathogenic strains of *M. circinelloides* [14].

Exogenously-induced RNA silencing in *M. circinelloides* is associated with the accumulation of two size classes of siRNAs, 21 and 25 nt long, which are differentially accumulated during the vegetative growth [15]. Only one of the two *dicer* genes that have been identified in *M. circinelloides*, *dcl-2*, is required for transgene-induced silencing and accumulation of the two size classes of siRNAs, both when silencing is induced by sense and inverted repeat transgenes [16,17]. Unlike *N. crassa*, RNA silencing in *M. circinelloides* is associated with an amplification step that generates secondary siRNAs corresponding to target sequences by the RNA-dependent RNA polymerase activity of the *rdrp-2* gene product [18]. A functionally distinct *rdrp* gene, *rdrp-1*, is essential for initiation of silencing by sense transgenes by producing antisense RNA transcripts derived from the transgene, but it is not involved in the amplification of the silencing signal [18]. Finally, only one of the three *ago* genes identified in *M. circinelloides*, *ago-1*, is required for transgene-induced gene silencing, whatever the nature of the silencing trigger is, as shown by the negative silencing phenotype of *ago-1*^*−*^ mutants expressing sense- or inverted-repeat transgenes. Since neither primary nor secondary siRNAs are detected in those mutants, it has been suggested that Ago-1 is required for production/stability of siRNAs [19].

As in metazoans, the *M. circinelloides* RNAi pathway also has a role in the regulation of endogenous genes through several classes of esRNA molecules, which are generated from genome-encoded precursors [20]. Deep sequencing of small RNAs endogenously accumulated in the wild type strain, *dicer*^*−*^ and *rdrp*^*−*^ mutants identified a number of esRNAs that map to exons and regulate the expression of many protein coding genes. These esRNAs, named exonic-siRNAs (ex-siRNAs), can be classified in different classes based on the silencing proteins required for their biogenesis. In addition to its role in silencing exogenous sequences, *ago-1* is also required for the production of all of these ex-siRNAs [19]. A large group of them (Class II), including 222 exons, is *dcl-2*-dependent and also requires the *rdrp-1* gene product, whereas a small group of only nine exons (Class I), which is also *dcl-2*-dependent, does not require the *rdrp-1* gene product but most of them requires RdRP-2 [20]. These two *dcl-2*-dependent classes of ex-siRNAs are down-regulated in the *ago-1*^*−*^ mutant and are specifically bound to Ago-1, suggesting that Ago-1 is involved in the biogenesis/stability of these regulatory ex-siRNAs. Binding to Ago-1 indicates that they are functional siRNAs produced by a canonical RNAi pathway to suppress the expression of the corresponding target genes. In fact, validation experiments demonstrated that lack of detection of specific ex-siRNAs of these classes in the *ago-1*^*−*^ mutant was associated with an increase of mRNA accumulation of the corresponding protein coding genes. Thus, these ex-siRNAs regulate the expression of the protein coding genes from which they are derived [19]. Classes III and IV of ex-siRNAs do not specifically bind Ago-1, although they are down-regulated in the *ago-1*^*−*^ mutant, suggesting that Ago-1 participates in the biogenesis of these ex-siRNAs [19]. Class III ex-siRNAs (88 exons) can be produced either by Dcl-1 or Dcl-2 and requires RdRP-1 and RdRP-2 for their biogenesis, whereas class IV is a tiny group of ex-siRNAs that requires the *dcl-1* gene product [20]. The biogenesis requirements and structural characteristics of classes III and IV have suggested that these ex-siRNAs are not produced by a canonical RNAi pathway, a frequent situation in filamentous fungi [21].

Knowledge of the endogenous functions regulated by esRNAs in fungi has been hampered by the lack of clear phenotypes in the majority of mutants affected in the RNAi machinery. However, *M. circinelloides* silencing mutants display diverse phenotypes [5]. The *dcl-1*^*−*^ mutants are affected in their development, showing a significant reduction in the vegetative growth rate and alterations in hyphal morphology, which are observed in the leading edge of the fungal colony [16]. Also, a light-regulated process as spore production is significantly reduced in *dcl-2*^*−*^, *dcl-1*^*−*^*/dcl-2*^*−*^, and *ago-1*^*−*^ mutants relative to the wild-type strain [17,19]. Besides a defect in sporulation, *ago-1*^*−*^ and other silencing mutants, such as *dcl-2*^*−*^ and *rdrp-2*^*−*^, share a common phenotype related to the autolytic response to nutritional stress [19]. The autolysis of filamentous fungi is not a simple cell necrosis phenomenon but is an active and well regulated process where many enzymatic activities are involved [22]. The accelerated lysis phenotype shared by these silencing mutants suggests that this process could be controlled by gene/s regulated by ex-siRNAs. Vegetative growth, hyphal morphology, sporulation and autolysis are developmental responses to endogenous and environmental signals in which many genes are involved, thus revealing the importance of the RNAi machinery in the endogenous regulation of complex developmental processes. The involvement of the RNA silencing pathways in the response to environmental signals is supported by the ability of *M. circinelloides* to adapt to the environment through RNAi-dependent epimutations [14], pointing out the relevance of the RNAi mechanism in controlling phenotypic plasticity.

We have previously demonstrated that *M. circinelloides* ex-siRNAs regulate the expression of the protein coding genes from which they are derived [19,20]. To have a comprehensive picture of genes regulated by the silencing machinery during the exponential and stationary growth phases, we have addressed the characterization of the mRNA profiles shown by mutants in the RNAi genes. In addition, we have achieved a more complete phenotypic characterization of silencing mutants, mainly on aspects related to the response to different environmental stimuli. The results obtained allow getting a deeper insight into the role of the RNAi machinery in the regulation of endogenous processes in *M. circinelloides*.

## Results

### Differential response to environmental signals of mutants in RNAi genes

**Figure 1:**
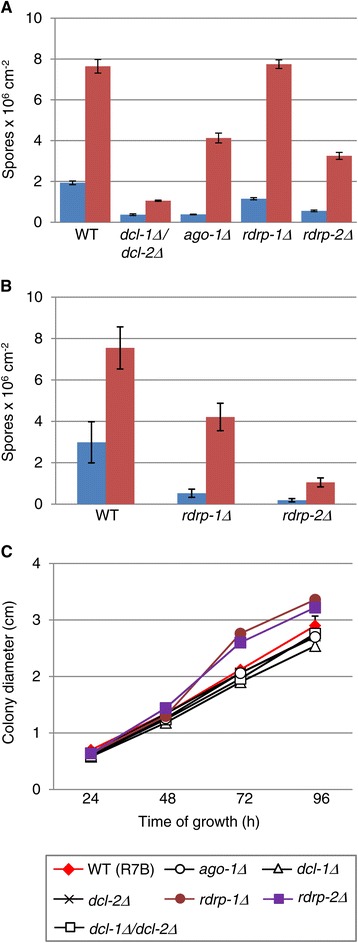
**Asexual spore production and growth in** ***rdrp-1***Δ **and** ***rdrp-2***Δ **mutants. A.** Asexual spores produced by wild-type strain (R7B) and the indicated mutants grown in the dark (blue bars) or in the light (red bars) for 72 h in solid MMC medium pH 3.2. **B**. Asexual spores produced by wild-type strain (R7B) and *rdrp-1*Δ and *rdrp-2*Δ mutants grown in the same conditions, except that solid YNB medium was used instead of MMC. The values are means and standard errors of 10 independent measurements. **C**. Radial growth of RNAi mutants at pH 2.25. A drop (5 μL) with 1000 spores of the indicated strains was deposited at the center of a Petri dish with solid complete medium (YPG) at pH 2.25. The radial growth was monitored periodically by measuring colony diameter. Averages and standard errors from five colonies per strain are shown.

Phenotypes of *dcl-2Δ* and *ago-1Δ M. circinelloide*s silencing mutants are consistent with the involvement of the RNAi machinery in processes dependent on environmental signals, such as asexual sporulation and autolysis [17,19]. The sporulation phenotype of *rdrp-1Δ* and *rdrp-2Δ* mutants shown in Figure 1A reinforces that involvement. In semi-complex medium only the *rdrp-2Δ* mutant showed a defect in the production of spores, which decreases at comparable levels of the *dcl-2Δ* and *ago-1Δ* mutant [17,19], whereas the spore production of the *rdrp-1Δ* strain was not significantly different to the wild-type strain. In minimal medium, however, both *rdrp*^*−*^ mutants showed a more defective sporulation (Figure 1B), pointing out the relevance of the environmental conditions for the mutant phenotypes.

**Figure 2:**
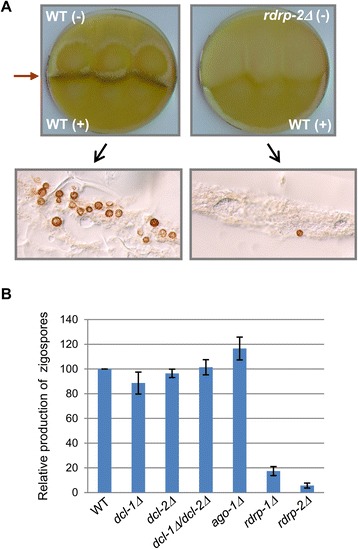
**Zygospore production of RNAi mutants. A**. Mating assays of the wild type strain NRRL3631 (+) with the (−) strains R7B (wild type, left) and *rdrp-2*Δ (right). Sexual interaction with formation of zygospores in the contacting zone (red arrow) can be only seen in the wild type cross. A histological section of the sexual interaction area observed under optical microscopy (10X) is shown. **B**. Relative zygospore production in genetic crosses between the indicated (−) strains and the wild-type NRRL3631 (+). Data are the averages and standard errors of the zygospores quantified in twelve histological sections (0.003 cm^2^) of the sexual interaction area for each cross after normalization against the zygospores produced in crosses between wild-type strains.

In addition to asexual sporulation and autolysis, other phenotypes related to responses to environmental signals were assessed in a double *dcl-1Δ*/*dcl-2Δ* knockout mutant and single knockout mutants for *ago-1*, *dcl-1*, *dcl-2*, *rdrp-1* and *rdrp-2*. Statistically significant differences were observed for two phenotypes: growth under low pH conditions and sexual interaction with opposite mating types. The ability of mutants to grow at different pH (2.25, 3.2, 4.5, 6.0, 7.0 and 8.0) was analyzed in complete YPG plates by measuring colony diameter every 24 h. Differences with respect to the wild-type strain (*t*-student analysis) were observed only under very low pH (2.25) for the *rdrp-1Δ* and *rdrp-2Δ* mutants, which showed a significant accelerated growth after 48 h of incubation under those conditions (Figure 1C) producing colonies of different size at 72 h (*P* <0.01). These mutants also exhibited a defect in the formation of zygospores, which are the structures produced as result of the sexual interaction between strains of opposite mating type, and where meiosis takes place. Compared with the wild type and the remaining silencing mutant strains, the *rdrp-1Δ* and *rdrp-2Δ* mutants produced significantly fewer zygospores (*t*-student analysis) when confronted with a strain of opposite mating type (Figure 2). Together, these results suggest that *rdrp-1* and *rdrp-2* should specifically regulate a group of genes involved in sexual interaction and formation of zygospores and in the ability to grow under extreme pH conditions, highlighting the involvement of the RNAi machinery in the response to environmental signals.

### Transcript profiles of the RNAi machinery mutants

Characterization of the mRNA profiles of mutants in the RNAi genes may result in the identification of genes involved in the phenotypic changes observed in such mutants. mRNA of the *M. circinelloides* wild-type and four mutant strains (*dcl-1Δ*/*dcl-2Δ*, *ago-1Δ*, *rdrp-1Δ* and *rdrp-2Δ*) was purified from mycelia grown during 24 h (exponential phase) and 48 h (stationary phase) on solid YPG medium and subjected to high-throughput sequencing. The rationale for using these conditions for transcriptome analysis is because they were the conditions in which the ex-siRNAs were characterized in previous studies [19,20], allowing us to connect mRNA accumulation values with the presence of a specific repertoire of ex-siRNA molecules. Besides that, these conditions would also allow us to link transcriptomic data with specific phenotypes, such as the defect in asexual sporulation shown by several silencing mutants, since this process is clearly evident at both exponential and stationary growth phases. The mRNA profiles for each mutant were compared with that of the wild-type strain at the same growth phase, considering as differentially expressed genes those that showed an adjusted p-value lower than 0.05 (false discovery rate of 0.05). The absence of any of the silencing genes produced significant alterations in the mRNA profiles of the fungus, especially in the exponential phase (Table 1, Additional file 1: Table S1). Although we identified up-regulated genes, as it would be expected from direct regulation by ex-siRNAs, the number of down-regulated genes in the mutants was higher than the up-regulated ones, predominantly during the exponential growth, indicating that the loss of function of RNAi components mainly results in repression instead of induction. As an average, differentially expressed genes showed at least a three-fold increase or decrease in mRNA accumulation in silencing mutants relative to the wild type (Table 1). Moreover, the level of repression or induction for a significant number of genes was very high, especially at exponential phase, with log_2_ fold change values higher than 3 or lower than −3 (Additional file 1: Table S1). Although the expression of some genes was altered at both 24 h and 48 h, most of them showed altered expression only in one of the analyzed growth phases (Additional file 1: Table S1), suggesting that different ex-siRNAs are produced during the vegetative growth. In most of the genes in which the expression was altered at both 24 h and 48 h, the regulation profile was very similar (Additional file 1: Table S1).

**Table 1:** **Genes differentially expressed in silencing mutants**

**Growth time (h)**	**Strain**	**Up-regulated genes**		**Down-regulated genes**	
		**Number**	**Average Log**_**2**_ **fold change** ^**a**^	**Number**	**Average Log**_**2**_ **fold change** ^**a**^
24	*dcl-1*Δ/*dcl-2*Δ	35	1.78	196	−3.13
	*ago-1*Δ	99	1.62	298	−3.00
	*rdrp-1*Δ	21	1.50	331	−3.00
	*rdrp-2*Δ	46	1.72	81	−2.43
48	*dcl-1*Δ/*dcl-2*Δ	25	2.34	49	−1.99
	*ago-1*Δ	46	2.15	86	−1.72
	*rdrp-1*Δ	7	2.10	26	−2.04
	*rdrp-2*Δ	53	1.72	54	−1.90

^a^Average values of the log_2_ fold change of regulated genes in the different *M. circinelloides* RNAi mutants compared to wild type. Log_2_ fold changes in Additional file 1: Table S1 were used to calculate the averages.

**Figure 3:**
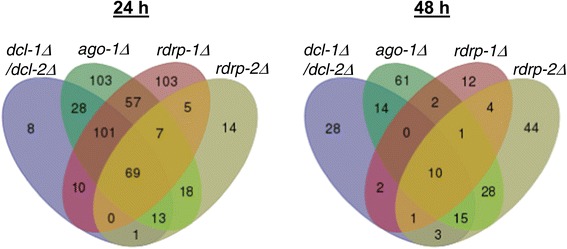
**Overlapping of genes regulated in RNAi mutants.** Four-way Venn diagram depicting the extent of overlap between the gene expression profiles at 24 h (left) and 48 h (right) from the indicated RNAi mutants. R7B was used as wild-type strain.

**Figure 4:**
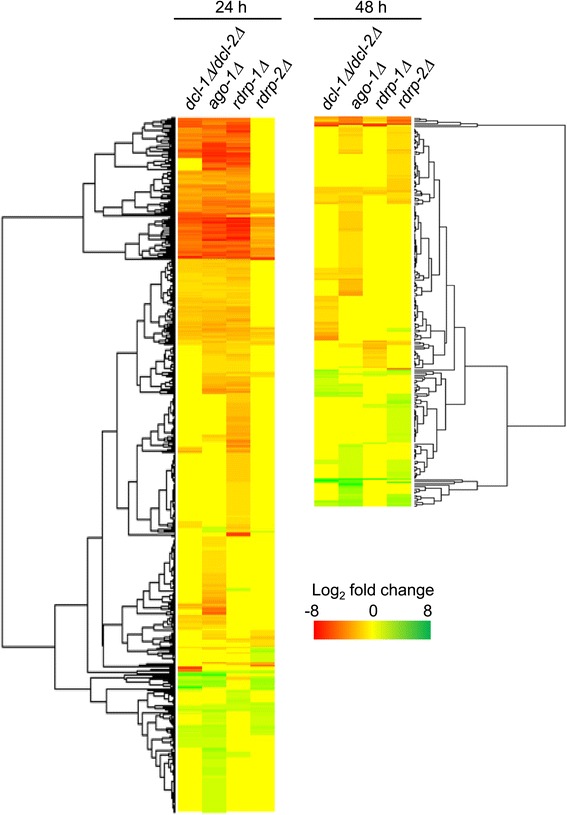
**Heat map depicting gene expression in RNAi mutants.** Hierarchical clustering of expression patterns of differently expressed genes in the indicated mutants at exponential phase (24 h of growth; left panel) and at stationary phase (48 h of growth; right panel) are shown. Red and green colors represent down- and up-regulation, respectively.

The comparison of genes differentially expressed in different mutants in relation to the wild-type strain revealed a high degree of coincidence, since most of the identified genes showed differential expression in more than one mutant (Figures 3 and 4). This coincidence was extended to the type of regulation (Figure 4). Thus, most of the genes up-regulated or down regulated in one mutant were regulated in the same way and with similar strength in other mutants in which their expression was affected (Figure 4, Additional file 1: Table S1). The comparison also showed preferential involvement of different combinations of silencing proteins depending on the type of regulation and the growth phase. Thus, 179 out of 196 genes (91.3%) that were repressed in the *dcl-1Δ/dcl-2Δ* double mutant at 24 h were also repressed in the *ago-1Δ*, and of these, 162 were also repressed in the *rdrp-1Δ* mutant (Figure 4, Additional file 1: Table S1). Conversely, *rdrp-2* plays a minor role in the regulation of these genes, since only 61 out of 179 (34.1%) genes differently expressed in *dcl-1Δ/dcl-2Δ* and *ago-1Δ* mutants were also repressed in the *rdrp-2Δ* strain, all of them being also altered in *rdrp-1Δ* (Figure 4, Additional file 1: Table S1). These results suggest an essential role of Dicer, Ago-1 and RdRP-1 proteins in the regulation of this gene cluster (Figure 4). Since these are the proteins required for the biosynthesis of class II ex-siRNAs, and given that the genes were down-regulated in the mutants instead of up-regulated, genes identified in this cluster could be secondary targets of this group of ex-siRNAs.

The role of *ago-1* in genes regulated by *dcl-1/dcl-2* was also observed in the up-regulated genes, since 32 out of 35 genes that increased their expression in the *dcl-1Δ/dcl-2Δ* mutant at 24 h showed the same behavior in the *ago-1Δ* strain. However, in this case, most of these genes (59.4%) were also up-regulated in the *rdrp-2Δ* strain (Figure 4, Additional file 1: Table S1) and only a small proportion of them (1%) also required *rdrp-1*, with a single gene showing increased expression exclusively in *dcl-1Δ/dcl-2Δ*, *ago-1Δ* and *rdrp-1Δ* (Figure 4, Additional file 1: Table S1). These results indicate that, besides Dicer and Ago-1, the RdRP-2 protein plays a prominent role in the up-regulation of genes during the exponential growth and suggest the participation of class I ex-siRNAs in this regulation, since those are the proteins required for the biogenesis of this ex-siRNA class.

The expression patterns observed at 48 h were very similar to those observed at 24 h, although the number of regulated genes was smaller (Figure 4, Table 1, Additional file 1: Table S1). However, there were some significant differences mainly concentrated in the repressed genes. Thus, the proportion of down-regulated genes in *dcl-1Δ/dcl-2Δ* that were also down-regulated in *ago-1Δ* was smaller (43.8%) than at 24 h and most of them (66.7%) were regulated by *rdrp-*2. This is in contrast to what happens at 24 h, where most of the repressed genes were regulated by *rdrp-1*. During stationary growth, only 38.1% of the genes repressed in *dcl-1Δ/dcl-2Δ* and *ago-1Δ* were also repressed in *rdrp-1Δ*, being all of them also altered in *rdrp-2Δ* (Figure 4, Additional file 1: Table S1). These results suggest that *rdrp-2* plays a more relevant role than *rdrp-1* in down regulation of genes during stationary phase (Figure 4, Additional file 1: Table S1). Moreover, changes in expression were not as large as those observed at exponential growth, with a small number of genes having Log_2_ fold change values lower than −3 (Table 1, Additional file 1: Table S1). On the other hand and similarly to what was observed at 24 h, most of genes (61.1%) induced in *dcl-1Δ/dcl-2Δ* and *ago-1Δ* mutants at 48 h were also induced in *rdrp-2Δ*, even at higher levels than in exponential phase (Table 1) and only a small proportion of them (11.1%) were also up-regulated in *rdrp-1Δ* (Figure 4, Additional file 1: Table S1). No genes showing increased expression exclusively in *dcl-1Δ/dcl-2Δ*, *ago-1Δ* and *rdrp-1Δ* were found (Figure 4, Additional file 1: Table S1). The major expression pattern among the up-regulated genes (that is, *dcl-1/2*, *ago-1* and *rdrp-2*-dependent) is expected from a direct regulation by ex-siRNAs of class I, as it is the case for two of the identified genes (ID115001, ID82197) (Additional file 1: Table S1), which correspond to class I ex-siRNA-producing loci [20]. Finally, an important proportion of genes (307 out of 537 at 24 h and 152 out of 225 at 48 h) were differentially expressed in one or more mutants but not in *dcl-1Δ/dcl-2Δ*, suggesting that they are regulated by a *dicer*-independent RNAi pathway ([20]; our unpublished results).

### Validation of RNA-seq data

**Figure 5:**
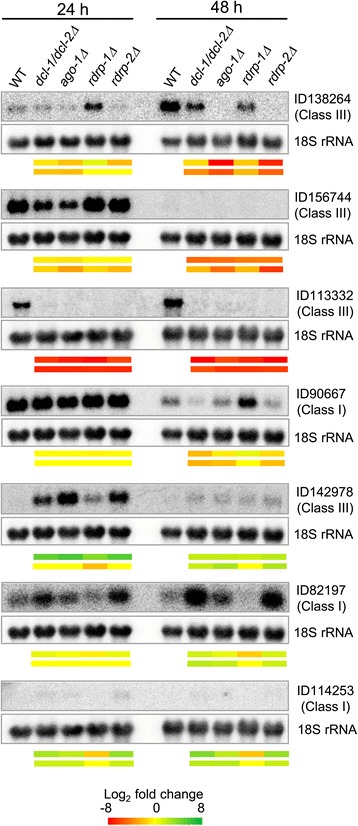
**Accumulation of mRNAs in wild type and mutant strains.** Northern blot analysis of total RNA (50 μg) isolated from the wild-type strain R7B and the indicated RNAi mutants grown for 24 h and 48 h in solid rich medium (YPG) pH 4.5. Membranes were hybridized with specific probes corresponding to the indicated genes (Additional file 3: Table S3) and reprobed with an 18S rRNA probe to check loading. Color bars depicted below each northern-blot show the log_2_ fold change in mutants versus wild-type strain at each time estimated from densitometric analysis of hybridization signals (upper bar) and RNA-seq analysis (lower bar).

As expected, the RNAi genes used in this study (*dcl-1, dcl-2*, *ago-1, rdrp-1* and *rdrp-2*), were all present in the pool of down-regulated genes identified in the corresponding RNAi mutant strains (Additional file 2: Table S2), thus constituting a good internal validation of the RNA-seq data. The reads corresponding to deleted genes detected in the knock-out mutants are expected, as those mutants contain partial deletions of the genes [16-19]. Additionally, a group of genes were selected based on their different patterns of significant differential expression among various mutants and the wild-type strain, or their putative function and potential interest for the phenotypes observed in the mutants (Additional file 2: Table S2). The expression of both up- and down-regulated genes in the mutants was analyzed at 24 h and 48 h of growth by northern-blot hybridization using specific probes (Additional file 3: Table S3). All the analyzed genes showed alterations in the accumulation of mRNA in RNAi mutants at 24 h or/and 48 h (Figure 5), indicating that they are under the control of the RNAi pathway. In fact, all down-regulated genes identified by RNA-seq data showed a decrease in mRNA accumulation in specific mutants, while genes selected because of their up-regulation accumulated more mRNA in RNAi mutants than in the wild type. In most cases, the expression patterns from northern-blot hybridizations of particular mutants fitted perfectly with those derived from RNA-seq, emphasizing the reliability of the RNA-seq data. Moreover, the expression patterns at 48 h of the genes analyzed was consistent with direct or indirect regulation of those genes by ex-siRNAs of different classes. Thus, several genes (ID90667, ID82197 and ID114253) could be regulated by class I ex-siRNAs, as they showed reduced or increased expression in *dcl-1Δ/dcl-2Δ*, *ago-1Δ* and *rdrp-2Δ* mutants but not in *rdrp-1Δ*, while others (ID138264, ID156744, ID113332 and ID 142978) changed their expression level in both *rdrp-1Δ* and *rdrp-2Δ* mutants, suggesting the participation of class III ex-siRNAs in their regulation (Figure 5; [20]).

### Putative functions of genes regulated by the RNAi genes

**Figure 6:**
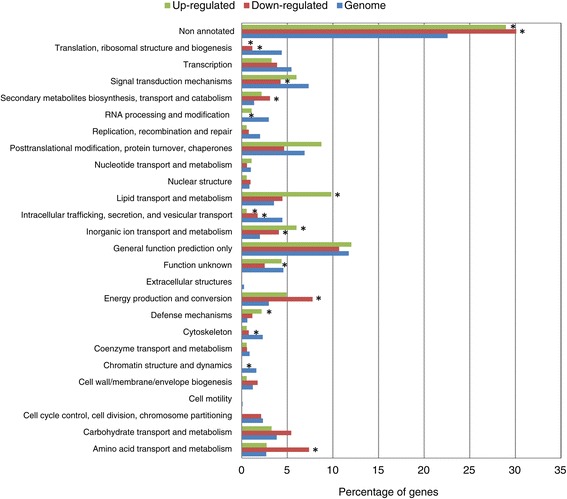
**Functional KOG class enrichment.** Bars represent the percentage of genes for each KOG class (*y*-axis) found in the genome (blue bars) and in down- (red bars) and up-regulated (green bars) genes in the silencing mutants. Differently expressed genes at exponential and stationary phases were joint. Asterisks indicate KOG classes showing significant differences in the down- or up-regulated genes relative to the total genome (*P* < 0.05; Pearson's chi-squared test with Yates' continuity correction).

To obtain an overview of the cellular processes controlled by the RNAi pathway during vegetative growth, enrichment analyses of EuKaryotic Orthologous Groups (KOG) terms within the “KOG class” category were carried out for up- and down-regulated genes in any silencing mutant at any time. The highest proportion of differentially expressed genes in the RNAi mutants corresponded to the non-annotated class, which was significantly enriched in up- and down-regulated genes (Figure 6). Within the KOG classes with known function, genes involved in processes such as translation, ribosomal biogenesis and structure, intracellular trafficking, secretion and vesicular transport were poorly represented among those regulated by the RNAi machinery components. However, others were severely affected in the RNAi mutants. KOG classes enriched in down-regulated genes included amino acid transport and metabolism, energy production and conversion, inorganic ion transport and metabolism, and secondary metabolites biosynthesis, transport and catabolism. On the other hand, KOG classes enriched in the up-regulated genes were inorganic ion transport and metabolism, defense mechanisms, and lipid transport and metabolism (Figure 6). The separated analysis of the regulated genes at 24 h and 48 h revealed a similar pattern of KOG class enrichment, although some differential details are relevant. Thus, carbohydrate transport and metabolism, and lipid transport and metabolism KOG classes were enriched in down- and up-regulated genes, respectively, only at 24 h, but not at 48 h (Additional file 4: Figure S1, Additional file 5: Figure S2). An enrichment in genes involved in carbohydrate transport and metabolism among those down-regulated in *dicer* mutants has been also reported in *Trichoderma atroviride* [23], where it has been suggested that the reduced expression of those genes could have a role in the growth and sporulation defects shown by the *dicer* mutants.

Analysis of the presumed functions of genes showing the highest differential expression in the RNAi mutants relative to wild-type identified genes that may be involved in growth, stress responses, and autolysis in *M. circinelloides* (Additional file 1: Table S1). Focusing on particular genes, we identified several down-regulated in RNAi mutants during exponential growth that code for proteins presumably involved in cell wall biogenesis and modification, including two putative chitin synthases (ID145794, ID104542) and four mannosyltransferases (ID14092, ID112156, ID 86104, ID 150168) (Additional file 1: Table S1), which are cell wall modifying proteins that are crucial for cell wall integrity and viability [24,25]. Also 10 genes involved in cell division were identified among the down-regulated ones, including 4 putative septins (ID156101, ID35281, ID104636, ID152534) (Additional file 1: Table S1). Down-regulation of those genes in RNAi mutants could be responsible for the phenotypic defects on growth and vegetative development shown by those mutants. Furthermore, some of the genes that were up-regulated in RNAi mutants during the exponential growth code for proteins containing domains associated with regulation of gene expression or signal transduction (Additional file 1: Table S1), which may explain the high number of deregulated genes in silencing mutants. Finally, several genes differently expressed at 48 h code for proteins probably related to the activity of transposons (ID113076, which contains a retrovirus zinc finger-like and retrotransposon gag protein domains), sexual interaction (ID107961, which codes for a short-chain dehydrogenase), sporulation, (ID156744, which is annotated as a carbohydrate esterase family 4 protein that deacetylates substrates such as chitin to chitosan, a component of the fungal cell wall/spore cortex), cellular aging (ID114253, a hydroxy-acyl-CoA dehydrogenase probably involved in cellular aging in rats [26]) and stress response (142978, a small heat-shock protein of the Hsp26/Hsp42 family) (Additional file 1: Table S1). Confirmation of the putative role of these and other genes on the different phenotypes shown by silencing mutants would require specific gene expression analysis under the condition of interest, in which the different phenotypes were clearly observed (see Discussion).

## Discussion

*M. circinelloides* represents an outstanding model for studying the function of the RNAi pathway in the control of physiological processes and development, since mutants in genes of this pathway show altered phenotypes, contrarily to what usually happens in other fungal models [27]. Phenotypes associated with mutations in genes involved in sRNA biogenesis have been described in few filamentous fungi, and in all cases the phenotypic changes have been moderate. Particularly, mutants in *dicer* genes of *Magnaporthe oryzae* and *T. atroviride* show a reduction in growth rate compared with the wild type [23,28]. In addition, the *T. atroviride* mutants are also affected in the production of vegetative spores [23]. The diverse phenotypes observed in the *M. circinelloides* RNAi mutants are probably a consequence of the altered expression of genes involved in different processes, since the ex-siRNAs generated by the RNAi pathway regulate the expression of a large number of protein coding genes [19,20]. Besides those from which they derive, these ex-siRNAs could also regulate in *trans* other genes with sequences completely or partially complementary to a particular ex-siRNA molecule. Given the vast number of ex-siRNAs with different sequences accumulated in the wild-type strain and the lack of knowledge about the required sequence complementarity, identification of all putative target genes of the ex-siRNAs by *in silico* analysis is an unaffordable task. One approach to identify genes controlled by the RNAi pathway that could be involved in fungal physiology and development is to compare the mRNA profiles of mutants in the RNAi pathway with that of the wild type strain. This work has used RNA-seq to characterize the mRNA profiles, at both exponential and stationary phase, of *dcl-1Δ/dcl-2Δ*, *ago-1Δ*, *rdrp-1Δ* and *rdrp-2Δ* mutants, which are affected in the key components of the RNAi pathway in *M. circinelloides* [17-19]. These conditions make possible to connect ex-siRNA classes produced during vegetative growth by specific combination of silencing proteins with gene clusters that show similar expression patterns in specific groups of silencing mutants, allowing the identification of ex-siRNA targets. Moreover, as production of asexual spores occurs during both exponential and stationary phase, the differential expression of specific genes in mutants affected in this process could support the role of those genes in the control of asexual sporulation.

Deletion of any of the silencing genes provoked a deep impact in mRNA accumulation at both growth phases, although the number of genes with altered expression was higher at exponential phase. In total, nearly 700 genes significantly changed their expression at least in one RNAi mutant and, interestingly, not only by increasing their mRNAs levels, as expected for a direct regulation by ex-siRNAs, but also by decreasing their expression. This suggests that the initial targets of ex-siRNAs can regulate the expression of other genes, which can be up- or down-regulated. In fact, only a small proportion (8.75%) of the genes up-regulated in the mutants corresponded to direct targets of ex-siRNAs, i.e. the ex-siRNAs-producing loci. This seems to be a common situation in fungi, since only a low proportion of up-regulated mRNAs in *T. atroviride dicer* mutants were correlated with down-regulation of the corresponding esRNAs [23]. However, the possibility that RNAi components could regulate gene expression by an unknown mechanism independent of ex-siRNAs cannot be excluded.

Expression of around 50% of the genes was altered in more than one mutant, indicating that several silencing proteins participate in the regulation of many genes. This is an expected result because ex-siRNAs are produced by different combination of silencing proteins, and therefore the mRNA accumulation patterns should replicate those of the ex-siRNA ones [20]. Dicer enzymes are involved in the biogenesis of the four classes of ex-siRNAs [20], and the mRNA accumulation patterns also revealed a main role for Dicer enzymes. However, the expression of a large number of genes was Dicer independent, supporting previous observations about the existence of a *dcl-1/dcl-2*-independent mechanism of ex-siRNA biogenesis [20], which is currently under study. On the other hand, almost all (86%) genes regulated by *dcl-1/dcl-2* are also regulated by *ago-1*, suggesting that the basic components of the canonical RNAi machinery are involved in this regulation (Figure 4).

A large proportion of the genes regulated by *dcl-1/dcl-2* and *ago-1* were also regulated by one or both *rdrp* genes at both exponential and stationary phase. The function of *rdrp* genes is particularly interesting, since their contribution to the regulation changes with the growth phase and the type of regulation. Most of the genes that are repressed in exponential phase in the *dcl-1Δ/dcl-2Δ* and *ago-1Δ* mutants were also repressed in the *rdrp-1Δ* mutant and may represent indirect targets of class II ex-siRNAs (*dcl-2*, *ago-1*, *rdrp-1*-dependent) (Figures 3 and 4). The class II ex-siRNAs derive mostly from genes coding proteins of unknown function which, in many cases, contain domains involved in signal transduction or in processing genetic information [20], and therefore, may act on a wide range of secondary response genes. A small proportion of the genes regulated by *dcl-1/dcl-2*, *ago-1* and *rdrp-1* were also regulated by *rdrp-2* and would represent secondary targets of class III (*dcl-1/dcl-2*, *ago-1*, *rdrp-1, rdrp-2*-dependent) or IV (*dcl-1*, *ago-1*, *rdrp-1, rdrp-2*-dependent) ex-siRNAs [19,20]. Class IV is a tiny group of ex-siRNAs that derive from only five exons and it is expected to have slight role in regulation, whereas class III ex-siRNAs derive from a large group of genes that are expressed at a high level in the wild type strain [19], which anticipates a significant role in controlling gene expression. The prevalent role of *rdrp-1* in down-regulation during exponential phase was not restricted to genes regulated by Dicer proteins but was also observed in Dicer-independent regulated genes (Figure 4).

The impact of *rdrp-1* in gene regulation seems to decrease with the growth phase, because most of the genes that are repressed during stationary phase in the *dcl-1Δ/dcl-2Δ* and *ago-1Δ* mutants were repressed in the *rdrp-2Δ* mutant but not in *rdrp-1Δ* (Figure 4). Only a few genes were down-regulated in the *rdrp-1Δ* mutant and all of them were also repressed in *rdrp-2Δ* (Figure 4). Therefore, most of genes down-regulated in *dcl-1Δ/dcl-2Δ* and *ago-1Δ* mutants at stationary phase could be indirect targets of class I ex-siRNAs, which is defined by their dependency on *dcl-2*, *ago-1* and, in most cases, *rdrp-2*. Besides that, some of those genes could be targets of classes III or IV, as it was confirmed by northern hybridization for three of them (ID138264, ID156744, ID113332) (Figure 5).

Contrary to what happens with the down-regulated genes, a similar expression pattern was observed for up-regulated genes at both exponential and stationary phase. Most of the genes with increased expression in the *dcl-1Δ/dcl-2Δ* and *ago-1Δ* mutants were also up-regulated in *rdrp-2Δ* both at exponential and stationary phase (Figure 4). Gene *rdrp-1* plays a minor role in the regulation of those genes, since only few of them increased their expression in both *rdrp-1Δ* and *rdrp-2Δ* mutants. These patterns suggest that they are direct targets of class I ex-siRNAs and, to a lesser extent, classes III or IV. The regulation pattern corresponding to these ex-siRNA classes was confirmed by northern-blot experiments (ID114253, ID82197, ID142978) (Figure 5).

Previous results and those of this work demonstrated that RNAi genes are involved in the control of several physiological and developmental processes, which include vegetative growth [16], production of asexual spores, accelerated autolysis in response to nutritional stress [17,19], growth at different pH conditions (Figure 1C) and sexual interaction (Figure 2). These processes are regulated by specific set of genes. Thus, asexual spore production is controlled mainly by *dcl-2*, *ago-1* and *rdrp-2*, with a possible role for *rdrp-1* gene in some particular conditions, such as poor nutrient environments. The same set of genes are also involved in accelerated autolysis [19], suggesting that both processes could be controlled by genes regulated directly or indirectly by ex-siRNAs, particularly class I, although regulation of those processes by RNAi components independently of ex-siRNAs cannot be excluded. In fungi, both cellular processes are connected with nutrient sensing of the cells, particularly carbon source levels in the medium [22,23]. Therefore, class I ex-siRNAs may be involved in fine tuning of genes involved in nutrient sensing pathways. Although only 5 different genes were identified as producers of this class of ex-siRNAs [20], the number of genes identified in our RNA-seq analysis that fit the class I ex-siRNA-dependent pattern was much higher, suggesting an amplification of the regulation. Thus, 26 genes showed mRNA patterns at 24 h, 48 h or at both growth times (ID114253 and ID77287) that correspond to regulation by class I ex-siRNAs (Additional file 6: Table S4). Interestingly, the mRNA levels of two of the 5 class I ex-siRNAs producer loci (ID82197 and ID115001) were increased at 48 h, one of them (ID82197) being validated by northern-blot hybridization (Figure 5). The identification of genes with a class I ex-siRNA expression pattern gives a strong support to the RNA-seq data and suggests that some of the identified genes may be involved in asexual spore production. This group of genes included a putative methyltransferase (ID90667) whose down-regulation in mutants with defective asexual sporulation was confirmed by Northern blot analysis (Figure 5). Methyltransferases have been shown to control several aspects of fungal development [29], including asexual sporulation in several fungal species [30-34]*.* In addition to this validated gene, other genes with similar expression pattern code for protein with putative functions or domains (Additional file 6: Table S4) that suggest that they could regulate sporulation. Thus, two genes (ID77287 and ID105388) code for proteins containing domains usually present in regulatory proteins, suggesting that they could be transcription factors controlling the expression of downstream genes. In addition, one gene (ID109934) codes for a predicted Rho GTPase-activating protein. Rho GTPases have been implicated in cellular integrity and spore wall maturation in *Schizosacharomyces pombe* [35,36], and the *M. circinelloides* Rho protein Cdc42 was found associated with chitosomes, which are the major reservoir of the chitin synthases required for the synthesis of the fungal cell wall [37]. Moreover, G proteins signaling pathways have been involved in sporulation in several fungi, such as *Aspergillus nidulans* and *N. crassa* [38]. Thus, modulation of the expression of this protein in the *dcl-2Δ*, *ago-1Δ* and *rdrp-2Δ* mutants could be responsible of the defects in sporulation shown by these mutants.

Other genes with the class I ex-siRNAs expression pattern (ID114253 and ID184709) are connected with lipid metabolism, which may play an important role in glucose starvation [23]. In particular, protein ID184709 is a putative lipase suggested to supply substrates for gluconeogenesis and energy production, a process necessary to support the morphological changes required for sporulation or to facilitate the energy storage in spores [23]. Taken together, the putative functions of several genes regulated by *dcl-2*, *ago-1* and *rdrp-2* genes, presumably through the action of class I ex-siRNAs, may explain the defects in asexual spore production shown by the corresponding null mutants. Detailed and individual functional analyses of each of those genes by deletion or overexpression would allow identification of the specific genes responsible for this phenotype.

The two processes regulated by RNAi identified in this work, growth at low pH and sexual interaction, are only controlled by *rdrp-1* and *rdrp-2*, suggesting that a different ex-siRNA class is involved. These ex-siRNAs would be produced by a Dicer-independent mechanism in which Ago-1 would not participate in their biogenesis or function, although other *M. circinelloides* Ago proteins (Ago-2 and/or Ago-3) could be involved. Alternatively, it can be also possible that RdRP-1 and RdRP-2 control these biological processes independently of the regulation by ex-siRNAs. The RNA-seq analysis revealed few genes regulated by both *rdrp-1* and *rdrp-2* during vegetative growth (Additional file 7: Table S5), although some of them could have a role in signal transduction (ID80421), gene regulation (ID164671), or post-translational modification (ID79209, an F-box domain protein). Further transcriptomic experiments performed under low pH conditions or during sexual interaction would probably shed light on *rdrp* regulation of these processes. Particularly interesting is the putative role of *rdrp* genes in the regulation of sexual interaction. However, efforts to analyze the transcriptomes of the wild type and mutant strains during sexual interaction have been hampered by the long time required for interaction to occur, which makes difficult to isolate the high quality RNA samples required for sequencing. In fact, RNA degradation starts several days before sexual interaction takes place*.* A similar problem has been found to identify differentially expressed genes with a possible role in the accelerated autolytic response shown by several silencing mutants, since autolysis induced by nutritional stress takes place after a long period of incubation [19]. Works are in progress to solve those problems and identify genes differentially expressed under the conditions of interest. Alternatively, detection of ex-siRNAs generated by an RdRP-dependent Dicer-independent RNAi pathway could enable the identification of target genes involved in the response to low pH and sexual interaction in *M. circinelloides*.

## Conclusions

Results obtained in this work reveal a role of canonical and non-canonical RNAi pathways in the regulation of a significant number of endogenous genes in *M. circinelloides* both at exponential and stationary growth phase. Cellular processes that respond to nutrient levels in the environment, such as autolysis and asexual sporulation, are regulated by the *dicer*-dependent RNAi pathway, whereas processes regulated by other signals, such as mating and low pH, are controlled by a non-canonical *dicer*-independent pathway. Several genes whose expression is modulated by each pathway code for proteins that could be involved in the regulation of specific physiological and developmental processes in response to environmental signals in this fungal model. Further analysis will identify the specific genes that are causative of the different phenotypes shown by the RNAi mutants.

## Methods

### Strains and growth conditions

The strains used in this study are listed in Additional file 8: Table S6. The leucine auxotroph R7B [39], derived from *M. circinelloides f. lusitanicus* CBS 277.49 [40], was used as the wild-type strain. All the mutant strains (Additional file 8: Table S6) derived from MU402, a uracil auxotroph derivative of R7B [16]. Cultures were grown in complete medium YPG [41], minimal YNB medium [42] or semi-complex MMC medium [16]. Media were supplemented with L-leucine (20 μg/ml) or uridine (200 μg/ml), when required. The pH was adjusted to 4.5 for mycelial growth. For RNA extraction, 2.5 · 10^5^ spores were grown at 26°C in solid YPG during 24 or 48 h under light conditions. Illumination conditions were as previously described [43].

### Quantification of sexual mating and zygospores formation

Sexual interactions were induced in complete yeast and dextrose agar (YPD) media, in which spores of the mutant strains ((−) mating type) were co-inoculated in the middle of the agar plates along with the wild type strain (NRRL3631, (+) mating type), placed approximately 2 cm apart. Plates were incubated at room temperature, under dark conditions during 20 days. Under these conditions, a dark line appears in the center of the plate as a consequence of the formation of zygospores in the contact zone. This line was sliced in portions of 1 cm^2^ and fixed in 10% formaldehyde during 10 hours. After fixation, samples were frozen and sliced using a cryotome to produce sections of 30 μm. Zygospores from twelve sections were counted by optical microscopy (bright field 10X) for each interaction.

### RNA extraction, library preparation and sequencing

Total RNA was extracted using RNeasy®Plant Mini Kit (Qiagen, Hilden, Germany) following the manufacturer instructions. In order to maximize target coverage, equal amounts of total RNA from three biological replicates of each strain were pooled for RNA-seq library construction. Both library preparation and sequencing was performed at Baseclear (Leiden, The Netherlands). The cDNA library was sequenced using Illumina HiSEQ2500 sequencer with a one lane, 50 cycles, single-read sequencing strategy. The total number of reads for each sample was between 12 and 21 million, more than 95% of them mapping onto the *M. circinelloides* genome (Additional file 9: Table S7).

### Validation of RNA-seq data by northern blot

Total RNA was isolated using Trizol reagent following the instruction of the supplier (Gibco-BRL). Standard recombinant DNA manipulations were performed as described in Sambrook and Russell [44] and Ausubel et al. [45]. Northern blots were hybridized to radioactively labelled probes and washed as described previously [15]. Probes of around 500 bp were generated by PCR amplification using specific primers for each gene (Additional file 3: Table S3). The PCR fragments were labelled with [α-^32^P]dCTP using Ready-to-Go DNA labelling beads (Amersham Pharmacia Biotech), following the instructions of the supplier. Membranes were exposed to Kodak Phosphor Screen SD230 for quantification. After exposure to the membrane, the screen was scanned on a Molecular Imager FX reader (BioRad) and signals were quantified using ImageJ software (National Institute of Health). The *25S* rRNA gene was used as an endogenous control to normalize for differences in the amount of RNA between samples [15].

### Sequence analysis

Gene based expression counts were calculated for annotated transcripts from the Joint Genome Institute (JGI) *M. circinelloides* genome using RSEM [46] and rounded to the nearest whole number. Expression values were then imported into R and differential expression analysis between wild-type and mutant samples was performed using DESeq [47]. To avoid infinite values, a value of 1 was added to the normalized count value of each gene with zero value before log_2_ transformation. Hierarchical clustering was performed using R function *hclust* with Manhattan distance.

### Availability of supporting data

The raw reads of *M. circinelloides* mRNA are deposited in the NCBI GenBank as Sequence Read Archive (SRA) under the following accession numbers: SRX731621 (wild type 24 h), SRX731623 (wild type 48 h), SRX731630 (*dcl-1Δ*/*dcl-2Δ* 24 h), SRX731637 (*dcl-1Δ*/*dcl-2Δ* 48 h), SRX731624 (*ago-1Δ* 24 h), SRX731629 (*ago-1Δ* 48 h), SRX731639 (*rdrp-1Δ* 24 h), SRX731640 (*rdrp-1Δ* 48 h), SRX731642 (*rdrp-2Δ* 24 h) and SRX731641 (*rdrp-2Δ* 48 h).

## Additional file

Additional file 1: Table S1. Genes differentially expressed in RNAi mutants *versus* wild-type strain. Normalized reads were used to calculate the Log_2_ fold changes in mRNA accumulation in each mutant compared to wild type strain. Differentially expressed genes were grouped in function of the number of mutants in which the expression was altered at 24 h or 48 h. Those genes with differential expression at both times were clustered according to their expression at 24 h. Within each cluster, genes were sorted for the fold change value in the left side mutant of each group. Decrease and increase in expression is shown in red and green, respectively, using the same scale as in Figure 4. Additional file 2: Table S2. Expression of RNAi genes and validated genes in RNAi mutants *versus* wild-type strain. Log_2_ fold change values were taken from Additional file 1: Table S1. Red and green colors represent down- and up-regulation, respectively, using the same scale as in Figure 4. Additional file 3: Table S3. Primers used to generate probes for mRNA validation experiments. Genes ID correspond to version 2.0 of *Mucor circinelloides* CBS277.49 genome. Italic and bold nucleotides were introduced to facilitate future cloning. Additional file 4: Figure S1. Functional KOG class enrichment of genes regulated by the RNAi machinery at exponential phase. Bars represent the percentage of genes for each KOG class (*y*-axis) found in the genome (blue bars) and in down- (red bars) and up-regulated (green bars) genes in the silencing mutants. Asterisks indicate KOG classes showing significant differences in the down- or up-regulated genes relative to the total genome (*P* < 0.05; Pearson's chi-squared test with Yates' continuity correction). Additional file 5: Figure S2. Functional KOG class enrichment of genes regulated by the RNAi machinery at stationary phase. Bars represent the percentage of genes for each KOG class (*y*-axis) found in the genome (blue bars) and in down- (red bars) and up-regulated (green bars) genes in the silencing mutants. Asterisks indicate KOG classes showing significant differences in the down- or up-regulated genes relative to the total genome (*P* < 0.05; Pearson's chi-squared test with Yates' continuity correction). Additional file 6: Table S4. Genes differentially expressed (Log_2_ fold change) showing a class I expression pattern (P < 0.05). Log_2_ fold change values were taken from Additional file 1: Table S1. Red and green colors represent down- and up-regulation, respectively, using the same scale as in Figure 4. Additional file 7: Table S5. Genes differentially expressed (Log_2_ fold change) exclusively in *rdrp-1*Δ and *rdrp-2*Δ mutants (*P* < 0.05). Log_2_ fold change values were taken from Additional file 1: Table S1. Red and green colors represent down- and up-regulation, respectively, using the same scale as in Figure 4. Additional file 8: Table S6. Strains used in this work. Additional file 9: Table S7. Sequencing data.
