# Supplementary material for: The RNAi machinery controls distinct responses to environmental signals in the basal fungus Mucor circinelloides
Source: BMC Genomics. 2015 Mar 25;16(1):237. doi: 10.1186/s12864-015-1443-2 (PMC4417260; doi:10.1186/s12864-015-1443-2)
Supplement: Additional file 2: Table S2. — Expression of RNAi genes and validated genes in RNAi mutants versus wild-type strain. Log2 fold change values were taken from Additional file 1: Table S1. Red and green colors represent down- and up-regulation, respectively, using the same scale as in Figure 4. [file 12864_2015_1443_MOESM2_ESM.docx]

| **Table S2.** Expression* of RNAi genes and validated genes in RNAi mutants *versus* wild-type strain | | | | | | | | | |
| --- | --- | --- | --- | --- | --- | --- | --- | --- | --- |
|  | 24 h | | | | 48 h | | | | Function |
| ID V2 | *dcl-1/2Δ* | *ago-1Δ* | *rdrp-1Δ* | *rdrp-2Δ* | *dcl1/2Δ* | *ago-1Δ* | *rdrp-1Δ* | *rdrp-2Δ* |  |
| 104148 | -1.8 |  |  |  | -2.0 |  |  |  | *dcl-1* |
| 104153 | -4.6 |  |  |  | -3.6 |  |  |  | *dcl-2* |
| 104161 |  | -4.8 |  |  |  | -3.4 |  |  | *ago-1* |
| 111871 |  |  | -2.2 |  |  |  | -1.6 |  | *rdrp-1* |
| 195368 |  |  |  | -5.3 |  |  |  | -4.2 | *rdrp-2* |
| 138264 | -1.7 | -1.8 |  |  | -3.2 | -4.8 | -1.9 | -4.4 | Predicted amino acid transporter |
| 156744 | -1.2 | -2.6 | -1.2 | -1.9 | -2.0 | -4.0 | -1.9 | -5.5 | Non-annotated |
| 113332 | -6.9 | -6.8 | -6.7 | -6.8 | -5.7 | -6.5 | -6.5 | -6.2 | Beta-1.6-N-acetylglucosaminyltransferase |
| 90667 |  |  |  |  | -2.1 | -1.8 |  | -1.8 | Methyltransferase |
| 142978 |  |  | -1.7 |  | 1.7 | 2.6 |  | 2.5 | Small heat-shock protein Hsp26/Hsp42 |
| 82197 |  |  |  |  | 2.1 | 1.8 |  | 1.6 | Non-annotated |
| 114253 | 1.6 | 1.6 |  | 1.7 | 2.1 | 2.2 |  | 1.7 | Short-chain alcohol dehydrogenase |

* Log_2_ fold change
