# Supplementary material for: The RNAi machinery controls distinct responses to environmental signals in the basal fungus Mucor circinelloides
Source: BMC Genomics. 2015 Mar 25;16(1):237. doi: 10.1186/s12864-015-1443-2 (PMC4417260; doi:10.1186/s12864-015-1443-2)
Supplement: Additional file 3: Table S3. — Primers used to generate probes for mRNA validation experiments. Genes ID correspond to version 2.0 of Mucor circinelloides CBS277.49 genome. Italic and bold nucleotides were introduced to facilitate future cloning. [file 12864_2015_1443_MOESM3_ESM.docx]

| **Table S3**. Primers used to generate probes for mRNA validation experiments | | |
| --- | --- | --- |
| **Gene ID** | **Primer name** | **Primer sequence*** |
| 138264 | 35121F | 5’- CAAT**CTCGAG**TATTGTAGTCTGGCTGGGCTGCT-3’ |
|  | 35121R | 5’-TACC**GAATTC**CACAACGAAGGATGTCATCTGGAC-3’ |
| 142978 | 39104F | 5’-CCAA**CTCGAG**TACAATACAGCTCGTCGCTCCT-3’ |
|  | 39104R | 5’-AGTT**GAATTC**ATGACAACCTTGAGCACGCCAT-3’ |
| 156744 | 50782F | 5’-ATTT**CTCGAG**ACCAGTCAGGCAAGGACACAA-3’ |
|  | 50782R | 5’-GCAC**GAATTC**ATCAGCACTTGGAGACCACA-3’ |
| 82197 | 82197F | 5’-CCTC**CTCGAG**TTTCCACAAGCCTCCAAG-3’ |
|  | 82197R | 5’-CGAT**GAATTC**TATTGCCCTTGGATCGCTTCGTAG-3’ |
| 164479 | 82942F | 5’-TTAG**CTCGAG**TCCTGAGCCTTTGCTCTCCTA-3’ |
|  | 82942R | 5’-AATA**GAATTC**AGAAACTGCGTTCCATCTCGTTC-3’ |
| 113332 | 83735F | 5’-TTCA**CTCGAG**GTATTATCTCGACCGTGAGC-3’ |
|  | 83735R | 5’-TGTT**GAATTC**AGTAGAGGTGGCAGATGATGAGG-3’ |
| 114253 | 84028F | 5’-CCTT**CTCGAG**GCATCCACAGCCCATATTCACT-3’ |
|  | 84028R | 5’-CAAC**GAATTC**GCCATCCAAGCCAAGTATAGACG-3’ |
| 90667 | 90667F | 5’-CCCA**CTCGAG**TGTGCGTCAGTGCAGTAGCCTTA-3’ |
|  | 90667R | 5’-CTTC**GAATTC**CGCTTTCCAGATTCCATGCC-3’ |
| * Italic and bold nucleotides were introduced to facilitate future cloning | | |
