# Supplementary material for: The RNAi machinery controls distinct responses to environmental signals in the basal fungus Mucor circinelloides
Source: BMC Genomics. 2015 Mar 25;16(1):237. doi: 10.1186/s12864-015-1443-2 (PMC4417260; doi:10.1186/s12864-015-1443-2)
Supplement: Additional file 4: Figure S1. — Functional KOG class enrichment of genes regulated by the RNAi machinery at exponential phase. Bars represent the percentage of genes for each KOG class (y-axis) found in the genome (blue bars) and in down- (red bars) and up-regulated (green bars) genes in the silencing mutants. Asterisks indicate KOG classes showing significant differences in the down- or up-regulated genes relative to the total genome (P < 0.05; Pearson's chi-squared test with Yates' continuity correction). [file 12864_2015_1443_MOESM4_ESM.pptx]

## Slide 1
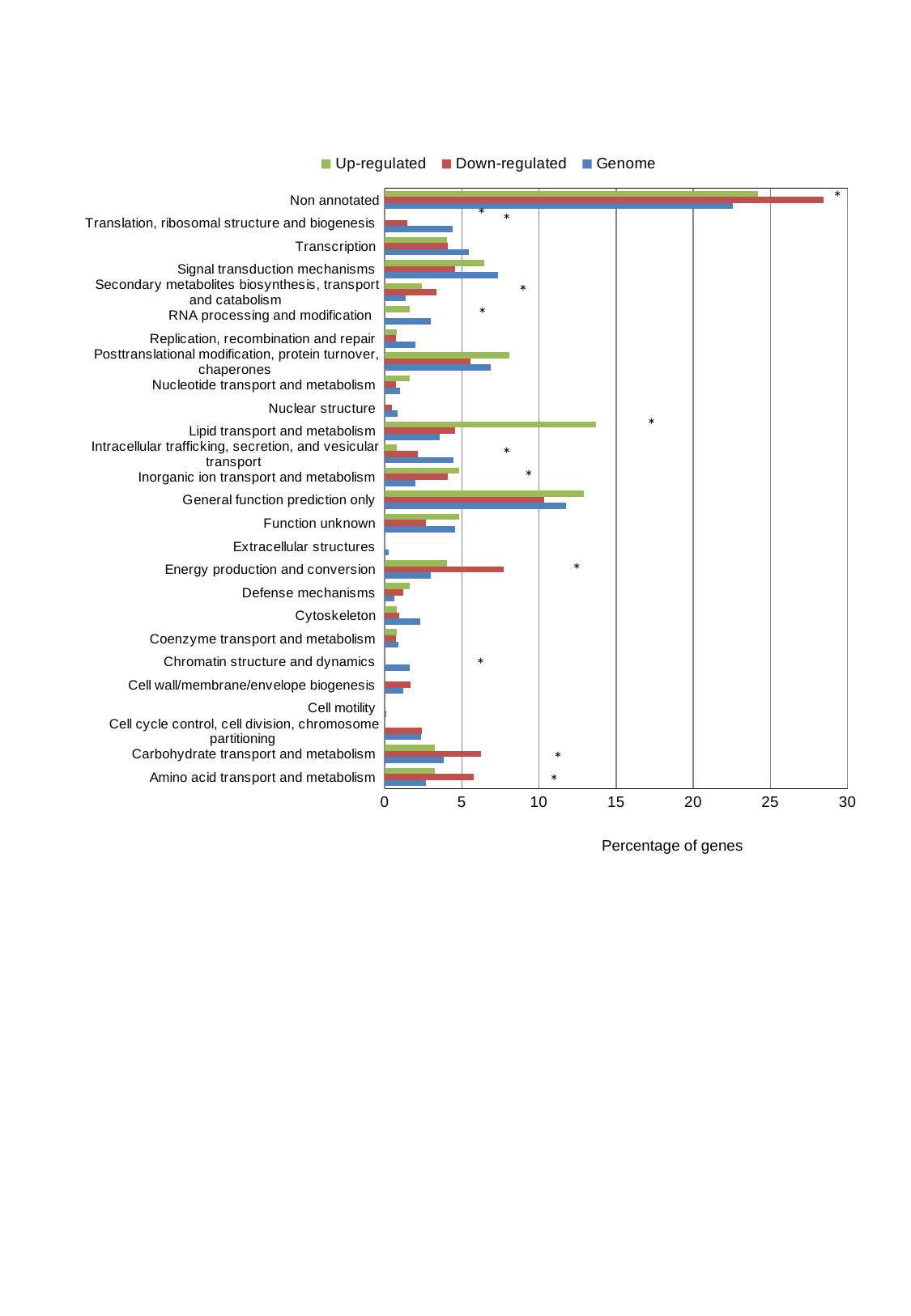

### Chart
| Category | | | |
|---|---|---|---|
| Amino acid transport and metabolism | 2.6879426572233127 | 5.783132530120482 | 3.225806451612903 |
| Carbohydrate transport and metabolism | 3.839918081747589 | 6.265060240963856 | 3.225806451612903 |
| Cell cycle control, cell division, chromosome partitioning | 2.3380834542196434 | 2.4096385542168677 | 0.0 |
| Cell motility | 0.09386466422049664 | 0.0 | 0.0 |
| Cell wall/membrane/envelope biogenesis | 1.220240634866456 | 1.6867469879518073 | 0.0 |
| Chromatin structure and dynamics | 1.6042324430412152 | 0.0 | 0.0 |
| Coenzyme transport and metabolism | 0.8703814318627869 | 0.7228915662650602 | 0.8064516129032258 |
| Cytoskeleton | 2.3039508490485536 | 0.9638554216867471 | 0.8064516129032258 |
| Defense mechanisms | 0.6229200443723868 | 1.2048192771084338 | 1.6129032258064515 |
| Energy production and conversion | 2.978069801177575 | 7.710843373493977 | 4.032258064516129 |
| Extracellular structures | 0.25599453878317263 | 0.0 | 0.0 |
| Function unknown | 4.5737690929260175 | 2.6506024096385543 | 4.838709677419355 |
| General function prediction only | 11.73308302756208 | 10.361445783132531 | 12.903225806451612 |
| Inorganic ion transport and metabolism | 2.005290553801519 | 4.096385542168675 | 4.838709677419355 |
| Intracellular trafficking, secretion, and vesicular transport | 4.462838126119975 | 2.1686746987951806 | 0.8064516129032258 |
| Lipid transport and metabolism | 3.541257786500555 | 4.578313253012048 | 13.709677419354838 |
| Nuclear structure | 0.8533151292772421 | 0.48192771084337355 | 0.0 |
| Nucleotide transport and metabolism | 1.0069118525471457 | 0.7228915662650602 | 1.6129032258064515 |
| Posttranslational modification, protein turnover, chaperones | 6.894786244560117 | 5.542168674698795 | 8.064516129032258 |
| Replication, recombination and repair | 2.0138237050942913 | 0.7228915662650602 | 0.8064516129032258 |
| RNA processing and modification | 2.9695366498848026 | 0.0 | 1.6129032258064515 |
| Secondary metabolites biosynthesis, transport and catabolism | 1.3653042068435874 | 3.3734939759036147 | 2.4193548387096775 |
| Signal transduction mechanisms | 7.338510111784282 | 4.578313253012048 | 6.451612903225806 |
| Transcription | 5.46121682737435 | 4.096385542168675 | 4.032258064516129 |
| Translation, ribosomal structure and biogenesis | 4.394572915777797 | 1.4457831325301205 | 0.0 |
| Non annotated | 22.570185169383052 | 28.433734939759038 | 24.193548387096776 |*
*
*
*
*
*
*
*
*
*
*
*
Percentage of genes
