# Supplementary material for: The RNAi machinery controls distinct responses to environmental signals in the basal fungus Mucor circinelloides
Source: BMC Genomics. 2015 Mar 25;16(1):237. doi: 10.1186/s12864-015-1443-2 (PMC4417260; doi:10.1186/s12864-015-1443-2)
Supplement: Additional file 5: Figure S2. — Functional KOG class enrichment of genes regulated by the RNAi machinery at stationary phase. Bars represent the percentage of genes for each KOG class (y-axis) found in the genome (blue bars) and in down- (red bars) and up-regulated (green bars) genes in the silencing mutants. Asterisks indicate KOG classes showing significant differences in the down- or up-regulated genes relative to the total genome (P < 0.05; Pearson's chi-squared test with Yates' continuity correction). [file 12864_2015_1443_MOESM5_ESM.pptx]

## Slide 1
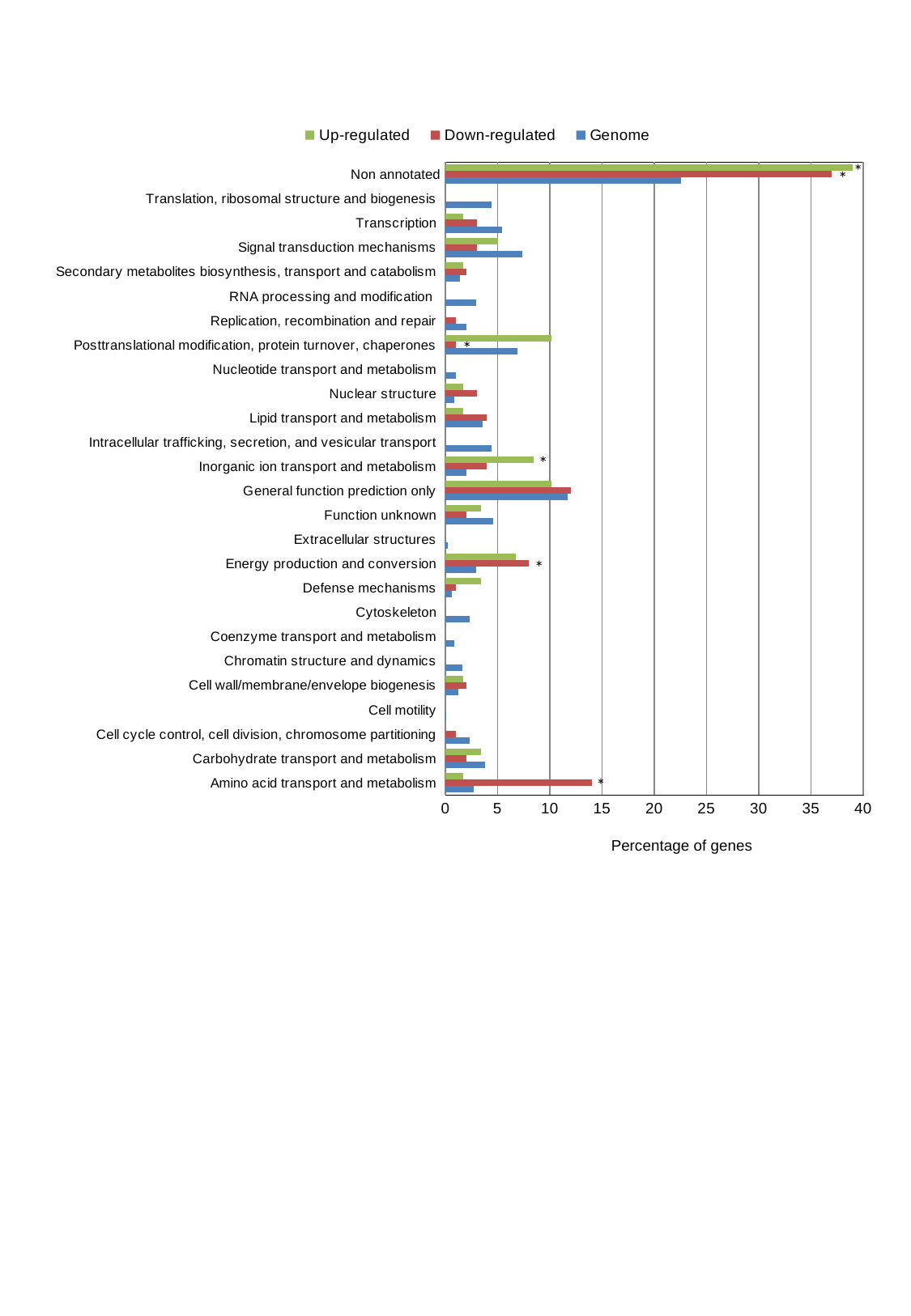

### Chart
| Category | | | |
|---|---|---|---|
| Amino acid transport and metabolism | 2.6879426572233136 | 14.000000000000002 | 1.694915254237288 |
| Carbohydrate transport and metabolism | 3.8399180817475886 | 2.0 | 3.3898305084745766 |
| Cell cycle control, cell division, chromosome partitioning | 2.338083454219643 | 1.0 | 0.0 |
| Cell motility | 0.09386466422049669 | 0.0 | 0.0 |
| Cell wall/membrane/envelope biogenesis | 1.220240634866456 | 2.0 | 1.694915254237288 |
| Chromatin structure and dynamics | 1.6042324430412156 | 0.0 | 0.0 |
| Coenzyme transport and metabolism | 0.8703814318627869 | 0.0 | 0.0 |
| Cytoskeleton | 2.3039508490485536 | 0.0 | 0.0 |
| Defense mechanisms | 0.6229200443723869 | 1.0 | 3.3898305084745766 |
| Energy production and conversion | 2.978069801177575 | 8.0 | 6.779661016949153 |
| Extracellular structures | 0.25599453878317263 | 0.0 | 0.0 |
| Function unknown | 4.573769092926019 | 2.0 | 3.3898305084745766 |
| General function prediction only | 11.733083027562078 | 12.0 | 10.169491525423732 |
| Inorganic ion transport and metabolism | 2.0052905538015193 | 4.0 | 8.474576271186441 |
| Intracellular trafficking, secretion, and vesicular transport | 4.4628381261199745 | 0.0 | 0.0 |
| Lipid transport and metabolism | 3.5412577865005552 | 4.0 | 1.694915254237288 |
| Nuclear structure | 0.853315129277242 | 3.0 | 1.694915254237288 |
| Nucleotide transport and metabolism | 1.0069118525471454 | 0.0 | 0.0 |
| Posttranslational modification, protein turnover, chaperones | 6.894786244560116 | 1.0 | 10.169491525423732 |
| Replication, recombination and repair | 2.0138237050942913 | 1.0 | 0.0 |
| RNA processing and modification | 2.9695366498848026 | 0.0 | 0.0 |
| Secondary metabolites biosynthesis, transport and catabolism | 1.3653042068435874 | 2.0 | 1.694915254237288 |
| Signal transduction mechanisms | 7.338510111784282 | 3.0 | 5.084745762711866 |
| Transcription | 5.46121682737435 | 3.0 | 1.694915254237288 |
| Translation, ribosomal structure and biogenesis | 4.394572915777796 | 0.0 | 0.0 |
| Non annotated | 22.570185169383056 | 37.0 | 38.983050847457626 |*
*
*
*
*
*
Percentage of genes
