# Supplementary material for: The RNAi machinery controls distinct responses to environmental signals in the basal fungus Mucor circinelloides
Source: BMC Genomics. 2015 Mar 25;16(1):237. doi: 10.1186/s12864-015-1443-2 (PMC4417260; doi:10.1186/s12864-015-1443-2)
Supplement: Additional file 8: Table S6. — Strains used in this work. [file 12864_2015_1443_MOESM8_ESM.docx]

| **Table S6**. Strains used in this work | | | |
| --- | --- | --- | --- |
| **Strain name** | **Genotype** | **Mating type** | **Reference or source** |
| R7B | *leuA1^-^* | (-) | [40] |
| NRRL3631 | Wild-type | (+) | NRRL collection |
| MU406 | *dcl-1Δ, leuA1^-^* | (+) | [16] |
| MU410 | *dcl-2Δ, leuA1^-^* | (+) | [17] |
| MU411 | *dcl-1Δ*/*dcl-2Δ* | (-) | [17] |
| MU413 | *ago-1Δ, leuA1^-^* | (-) | [19] |
| MU419 | *rdrp-1Δ, leuA1^-^* | (-) | [18] |
| MU420 | *rdrp-2Δ, leuA1^-^* | (-) | [18] |
