# Supplementary material for: The RNAi machinery controls distinct responses to environmental signals in the basal fungus Mucor circinelloides
Source: BMC Genomics. 2015 Mar 25;16(1):237. doi: 10.1186/s12864-015-1443-2 (PMC4417260; doi:10.1186/s12864-015-1443-2)
Supplement: Additional file 9: Table S7. — Sequencing data. [file 12864_2015_1443_MOESM9_ESM.docx]

**Table S7. Sequencing data**

| **Growth time (h)** | **Strain** | **Total number of reads** | **Sample yield (in MB)** | **Number of mapped reads^a^** | **Mapped reads (in percentage)** |
| --- | --- | --- | --- | --- | --- |
| 24 | WT (R7B) | 17,840,327 | 909 | 17,416,349 | 97.6 |
|  | *dcl-1Δ*/ *dcl-2Δ* | 18,878,811 | 962 | 18,394,820 | 97.4 |
|  | *ago-1Δ* | 21,389,732 | 1,090 | 20,861,035 | 97.5 |
|  | *rdrp-1Δ* | 17,037,660 | 868 | 16,285,254 | 95.6 |
|  | *rdrp-2Δ* | 15,760,260 | 803 | 15,433,959 | 97.9 |
| 48 | WT (R7B) | 15,162,239 | 773 | 14,791,685 | 97.6 |
|  | *dcl-1Δ*/ *dcl-2Δ* | 15,765,254 | 804 | 15,383,055 | 97.6 |
|  | *ago-1Δ* | 18,416,564 | 939 | 17,836,162 | 96.8 |
|  | *rdrp-1Δ* | 14,178,203 | 723 | 13,825,166 | 97.5 |
|  | *rdrp-2Δ* | 12,008,722 | 612 | 11,745,525 | 97.8 |

^a^Raw reads were mapped against *Mucor circinelloides* genome at JGI.
